# Dose–Response Relationship Between Physical Activity and the Incidence of Peripheral Artery Disease in General Population: Insights From the National Health and Nutrition Examination Survey 1999–2004

**DOI:** 10.3389/fcvm.2021.730508

**Published:** 2021-10-15

**Authors:** Cheng-Jia Qu, Le-Qun Teng, Xin-Nong Liu, Yong-Bao Zhang, Jie Fang, Chen-Yang Shen

**Affiliations:** ^1^Vascular Surgery Center, Chinese Academy of Medical Sciences, Peking Union Medical University Fuwai Hospital, Beijing, China; ^2^Vascular Surgery Department, Peking Union Medical College, Beijing, China

**Keywords:** peripheral artery disease, physical activity, ankle–brachial index, metabolic equivalents, dose–response relationship

## Abstract

**Purpose:** A low ABI, ≦0.9, indicates peripheral artery disease (PAD) and physical activity (PA) represents an important non-surgical treatment for patients with PAD. However, as for the general population, the associations between PA, PAD, and their mutual dependence are not well-defined. Here we aimed to determine whether there is a dose–response relationship between PA and incidence of PAD in the general population using restricted cubic spline (RCS).

**Patients and methods:** This study analyzed 1,370 adults aged ≧40 years who had participated in the National Health and Nutrition Examination Survey (NHANES) during 1999–2004. The ABI of the participants were measured by trained technicians, and PAD was defined as ABI ≦0.9. PA was obtained with a standard questionnaire, and metabolic equivalents (MET) were used to quantify the PA level. Logistic regression was used to assess the association between PA and incidence of PAD, and the dose–response relationship was analyzed with RCS.

**Results:** PAD was present in 6.2% of the participants: 5.6% of males and 6.9% of females. After adjusting for potential confounders, compared with the first quartile (Q1) of MET, the odds ratios (ORs) of PAD for those with Q2, Q3, and Q4 of MET were 0.688 [95% confidence interval (CI) = 0.684–0.692], 0.463 (95% CI = 0.460–0.466), 0.816 (95% CI = 0.812–0.821), respectively (all *p* < 0.0001). The RCS regression showed that physical activity was related to the incidence of PAD in a non-linear manner (*p* for non-linearity < 0.0001). For females, the prevalence of PAD decreased as physical activity increased, reaching the minimum for activity at ~5,800 MET-min month^−1^ (OR = 0.425, 95% CI = 0.424–0.426), and for males, no plateau was found in this study.

**Conclusion:** The prevalence of PAD is inversely associated with PA, and vigorous activities might help decrease PAD risk for general population. The prevalence of PAD reaches the minimum at ~5,800 MET-min month^−1^, representing a recommended PA value.

## Introduction

Peripheral artery disease (PAD) is more unrecognized compared with other cardiovascular diseases and, yet, is the most common cause of tissue loss, infection, and major amputation (1). It is reported that more than 200 million people have PAD worldwide (2). More than half of the patients diagnosed with PAD are asymptomatic (3). Despite the advanced nature of medical systems in many countries, the health hazard and economic burden of PAD remain high due to the requirement for frequent intervention (4). Thus, determining the etiology of PAD and taking effective measures are very important to reduce the burden of this condition. The ankle–brachial index (ABI) is the ratio of ankle to arm systolic pressure and a valid non-invasive measure that predicts for PAD as well as other cardiovascular events. Generally, ABI is among 1.0–1.4 in healthy individuals, and a low ABI (≦0.9) indicates PAD (5).

Though there is good evidence for higher physical activity (PA) levels reducing risk of cardiovascular disease (6), and exercise therapy is recommended for patients with intermittent claudication in the latest European Society of Cardiology guidelines for PAD (7), little is known about the association between the quantity, intensity of PA, and the incidence of PAD. Besides, most of the studies included in the guidelines are based on patients with intermittent claudication (8–10). The situation for the general population is not clear, and the dose–response relationship between PA and the incidence of PAD has not been reported before.

We hypothesized that there was an inverse dose–response relationship between PA and the incidence of PAD. This study aimed to determine whether there is a dose–response relationship between PA and the incidence of PAD in American adults by analyzing the data from the National Health and Nutrition Examination Survey (NHANES) from 1999 to 2004.

## Materials and Methods

### Study Design and Population

The data of this study were obtained from the NHANES 1999–2004, which is a large, multilevel, cluster probability sampling survey that collects health examination data from the non-institutionalized population in the USA. Beginning in 1999, NHANES was conducted as annual surveys, with data being released every 2 years. A more detailed information can be found at https://wwwn.cdc.gov/nchs/nhanes.htm. In the present study, subjects with missing values of ABI, blood glucose, total cholesterol, and other interested covariates were excluded from the analyses. To minimize the potential confounding from prior morbidity, we excluded participants with low cardiovascular fitness level (11). Finally, three consecutive NHANES cycles (1999–2000, 2001–2002, and 2003–2004, *N* = 31,126) were analyzed, and a total of 1,370 participants older than 40 years with complete date were included in our analysis. The available data had been weighed demographically, and the NHANES study protocol was approved by the National Center for Health Statistics ethics review board with all participants providing written informed consent.

### Outcome Variable

Briefly, the subjects lie supine on the exam table, and their systolic pressure are measured on the right arm (brachial artery) and both ankles (posterior tibial arteries) (12). If the participant has a rash or open wound on the right arm, dialysis shunt, right-sided radical mastectomy or any other condition that would interfere with accurate measurement or would cause discomfort to the participant, the left arm is used for the brachial pressure measurement. Systolic blood pressure is measured twice at each site for participants aged 40–59 years and once at each site for participants aged 60 years and older. If a health technician is unable to obtain a reading at a site, they may attempt another reading at the same site after a brief resting period. PAD was defined as ABI ≦0.9.

### Exposure Variable: Physical Activity

Each participant completed a PA questionnaire that was based on the Global Physical Activity Questionnaire (GPAQ) (13). The questionnaire collected the type, frequency (number of days per month), and duration (amount of time spent on a typical day) of PA in the past 30 days for a minimum of 10 min, including moderate- and vigorous-intensity recreational activities, moderate- and vigorous-intensity walking/bicycling for transportation and work. Vigorous-intensity activities were activities that cause significant increases in breathing or heart rate, while moderate-intensity activities were those that cause mild increase in breathing or heart rare. Work-related PA referred to paid or unpaid work, household chores, and yard work. Recreational PA referred to sports, fitness, and recreational activities. For the active transportation domain, information was collected on walking and bicycling activities.

Metabolic equivalent (MET) is a unit that describes the energy consumption while performing a specific activity and 1 MET is equal to 3.5 ml O_2_ kg^−1^ min^−1^. NHANES suggested that MET for vigorous work-related activity was 8.0, moderate work-related activity 4.0, walking or bicycling for transportation 4.0, vigorous leisure-time PA 8.0 and moderate leisure-time PA 4.0, respectively (14). For each activity, PA was calculated by multiplying the number of days by the mean duration by the recommended MET and then summed the values to obtain a value of total PA.

### Potential Confounding Factors

The basic characteristics of the involved subjects, including age (categorized into 40–64, 65–75, 75–85, and ≧85 years), gender (male or female), race (Mexican American, non-Hispanic White, non-Hispanic Black, or other race), socioeconomic characteristics including education level (<9th grade, 9–11th grade, high school graduate, some college, or AA degree or above), marital status (married/living with partner, widowed/divorced/separated, or never married), family poverty-to-income ratio (PIR ≦ 100% or >100%, PIR ≦ 100% indicates poverty), body mass index (BMI <18.5, 18.5 to <25.0, 25.0 to <30.0, or <30 kg m^−2^), were all analyzed to control the bias. In addition, the risk factors for PAD such as smoking (yes or no), drinking alcohol (yes or no), blood total cholesterol, and plasma fasting glucose were also homogenized. Except for BMI (15), blood total cholesterol (16), and plasma fasting glucose (17), all covariates were self-reported. BMI data were measured by NHANES technicians. For plasma fasting glucose test, participants were examined in the morning after fasting at least 8 h or more but <24 h. Blood specimens are processed, stored, and shipped to the University of Missouri—Columbia for analysis. For total cholesterol test, vials were shipped to the Johns Hopkins University Lipoprotein Analytical Lab for testing. Detailed specimen collection and processing instructions are presented in the NHANES Laboratory/Medical Technologists Procedures Manual.

### Statistical Analysis

According to the NHANES analytic guidelines (18), sampling weight was incorporated to account for its complex design. Data are presented as mean with SD or median with interquartile range (IQR) according to their distributions. Categorical variables were presented as frequency. Student's *t*-test or Mann–Whitney test were applied to compare continuous variates between groups. Chi-square test was employed to detect differences in categorical variates between groups. Rank-sum test was utilized to compare ordinal categorical variates between groups. Multivariate logistic regressions were conducted to explore the independent relationship between PA and ABI values, while adjusting for potential confounders. A trend test was used to observe linear trends between PA and the incidence of PAD, which is described previously (19).

RCS were used to detect the dose–response relationship between PA and ABI values, using the 25, 50, and 75th percentiles of the distribution of PA as fixed knots. Non-linear relationship between PA and the incidence of PAD was examined using RCS models. Non-linearity was tested for using likelihood ratio test to compare two models: one including only a linear effect and the second also including cubic spline terms (20, 21). The RCS models were adjusted for age, sex, race, education level, marital status, family PIR, health insurance, smoking status, drinking-alcohol status, BMI, total cholesterol, and plasma fasting glucose. Analyses stratified by sex were conducted. R software version 3.53 (R Foundation for Statistical Computing, Vienna, Austria) and SAS 9.4 (Cary, NC) were used for the analyses, and *p* < 0.05 was considered statistically significant.

## Results

### Baseline Characteristics

The prevalence of PAD was 6.2% of all individuals, 5.6% for male and 6.9% for female. The median MET were lower in participants with PAD than that without PAD (2,100.0 vs. 3,652.5 *p* < 0.05). The distribution of variables of interest is listed in Table 1. The incidence of PAD is significantly associated with age (*p* < 0.0001), education level (*p* < 0.05), smoking (*p* < 0.01), and plasma fasting glucose (*p* < 0.01). Overall, subjects with PAD were more likely to be older, widowed/separated/divorced, and to have a lower education level, a higher smoking proportion, and higher fasting plasma glucose level. However, there was no statistical differences in gender, race, family PIR, total cholesterol, and drinking alcohol between groups (all *p* > 0.05).

**Table 1:** Baseline characteristics of included participants.

**Characteristic**	**Non-PAD**	**PAD**	***Z/x^**2**^***	***P***
N (participants)	1,285	85		
MET, median (IQR)	3,652.5 (5,825)	2,100 (4,095)	−3.090	0.002^*^
Vigorous / Moderate, median (IQR)	0.00 (0.71)	0 (0.00)	−3.423	0.001^*^
Left ABI, median (IQR)	1.14 (0.13)	0.87 (0.18)	−12.687	<0.001^*^
Right ABI, median (IQR)	1.14 (0.14)	0.84 (0.17)	−14.358	<0.001^*^
Sex (%)			0.983	0.321
Male	691 (53.77)	41 (48.24)		
Female	594 (46.23)	44 (51.76)		
Age (%)			70.343	<0.001^*^
40–64	854 (66.46)	28 (32.94)		
65–74	255 (19.84)	21 (24.71)		
75–84	154 (11.98)	26 (30.59)		
≥85	22 (1.72)	10 (11.76)		
Race (%)			0.750	0.861
Mexican American	199 (15.49)	11 (12.94)		
Non-Hispanic White	849 (66.06)	57 (67.06)		
Non-Hispanic Black	167 (13.00)	13 (15.29)		
Other Race	70 (5.45)	4 (4.71)		
Education Level (%)			12.064	0.017^*^
<9th grade	116 (9.03)	15 (17.65)		
9–11th grade	132 (10.27)	11 (12.94)		
High school graduate	293 (22.80)	23 (27.06)		
Some college or AA degree	371 (28.87)	22 (25.88)		
College graduate or above	373 (29.03)	14 (16.47)		
Marital status (%)			5.010	0.082
Married/living with partner	924 (71.91)	52 (61.18)		
Widowed/divorced/separated	302 (23.50)	29 (34.12)		
Never married	59 (4.59)	4 (4.70)		
Family PIR			0.449	0.503
≤ 100%	109 (8.48)	9 (10.59)		
>100%	1176 (91.52)	76 (89.41)		
BMI, kg m^−2^ (%)			2.800	0.424
<18.5	11 (0.86)	2 (2.35)		
18.5– <25.0	359 (27.94)	22 (25.88)		
25.0– <30.0	533 (41.48)	32 (37.65)		
≥30.0	382 (29.72)	29 (34.12)		
Smoking (%)			8.216	0.004^*^
No	614 (47.78)	27 (31.76)		
Yes	671 (52.22)	58 (68.24)		
Drinking alcohol (%)			2.747	0.097
No	347 (27.00)	30 (35.29)		
Yes	938 (73.00)	55 (64.71)		
Total cholesterol, mg/dL, median (IQR)	206 (49.00)	205 (47.00)	−0.491	0.623
Glucose, plasma, mg/dL, median (IQR)	99.1 (17.10)	105.2 (19.80)	3.025	0.003^*^

*PAD, peripheral artery disease; MET, metabolic equivalents; PIR, poverty-to-income ratio; BMI, body mass index; IQR, interquartile range*.

* *Considered statistically significant p < 0.05*.

### Univariate and Multivariate Logistic Regression Analyses of the Relationship Between Physical Activity and Incidence of Peripheral Artery Disease

The distribution of MET quartiles is listed in Table 2, and the odds ratio (OR) and 95% CIs of the MET quartiles for PAD are listed in Table 3. The participants were divided into four groups according to the calculated MET. The first quartile (Q1) participants were used as a reference group (OR values = 1.0). The univariate analysis showed that compared with the Q1 of MET, with the increase in MET, the ORs of PAD for those with Q2, Q3, and Q4 of MET were 0.618 (95% CI = 0.614–0.621), 0.451 (95% CI = 0.449–0.454), and 0.597 (95% CI = 0.594–0.600), respectively (all *p* < 0.0001). The results indicated that, with the increase in PA, the likelihood of PAD decreased more than 50% for Q3 participants and 40% for Q4 participants.

**Table 2:** Distribution of MET quartiles and patterns of physical activities for included participants.

**Characteristic**	**Non-PAD**	**PAD**	***x^**2**^***	***P***
MET quartiles			9.567	0.023^*^
Q1	310 (24.12)	32 (37.65)		
Q2	321 (24.98)	21 (24.71)		
Q3	323 (25.14)	19 (22.35)		
Q4	331 (25.76)	13 (15.29)		
Vigorous/Moderate			12.640	<0.001^*^
=0	866 (67.39)	73 (85.88)		
>0	419 (32.61)	12 (14.12)		

*PAD, peripheral artery disease; MET, metabolic equivalents; SD, standard division*.

* *Considered statistically significant p < 0.05*.

**Table 3:** Odds ratio (OR) and 95% CIs of the MET quartiles for PAD.

**Variable**	**Univariate**		**Model I** ^**a**^		**Model II** ^**b**^	
	**OR (95%CI)**	***P*-value**	**OR (95%CI)**	***P*-value**	**OR (95%CI)**	***P*-value**
METs quartiles						
Q1	1		1		1	
Q2	0.618 (0.614–0.621)	<0.001^*^	0.636 (0.632–0.639)	<0.001^*^	0.688 (0.684–0.692)	<0.001^*^
Q3	0.451 (0.449–0.454)	<0.001^*^	0.437 (0.434–0.439)	<0.001^*^	0.463 (0.460–0.466)	<0.001^*^
Q4	0.597 (0.594–0.6)	<0.001^*^	0.735 (0.731–0.739)	<0.001^*^	0.816 (0.812–0.821)	<0.001^*^
*P* for trend		<0.001^*^		<0.001^*^		<0.001^*^

a *Adjust for age, sex, race, education level, and marital status*.

b *Adjust for age, sex, race, education level, marital status, family PIR, smoking, drinking alcohol, BMI, total cholesterol, and fasting plasma glucose*.

*PAD, peripheral artery disease; OR, odds ratio; OR <1.0 means lower likelihood for PAD*.

*CI, confidence interval; PIR, poverty-to-income ratio; BMI, body mass index*.

*METs increases from Q1 to Q4*.

* *Considered statistically significant p < 0.05*.

Then we included age, sex, education level, marital status, family PIR, smoking, drinking alcohol, BMI, total cholesterol, and fasting plasma glucose into the model. After adjusting for these confounding factors, the odds ratio for those with Q2, Q3, and Q4 of MET compared with Q1 of MET were 0.688 (95% CI = 0.684–0.692), 0.463 (95% CI = 0.460–0.466), 0.816 (95% CI = 0.812–0.821), respectively (all *p* < 0.001).

### Dose–Response Relationship Between Physical Activity and Incidence of Peripheral Artery Disease

**Figure 1:**
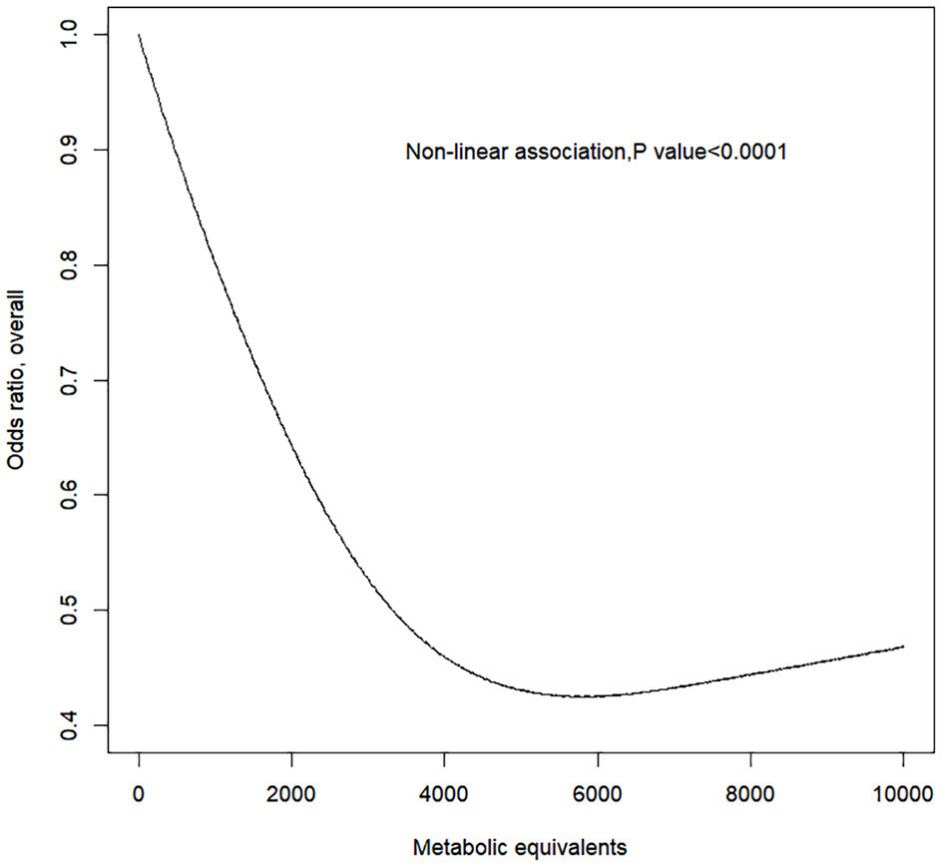
Response between physical activity and the prevalence of peripheral artery disease for all subjects. Adjusted cubic spline models showing association between physical activity and the prevalence of peripheral artery disease in all participants. Models are adjusted for age, gender, race, education level, marital status, family poverty-to-income ratio, smoking, alcohol, body mass index, total cholesterol, and fasting plasma glucose. Knots are the 25, 50, and 75th percentiles for metabolic equivalents.

**Figure 2:**
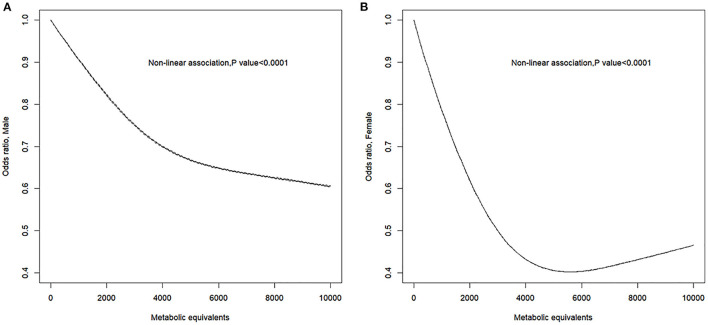
Sex-specific response between physical activity and the prevalence of peripheral artery disease. Adjusted cubic spline models showing association between physical activity and the prevalence of peripheral artery disease in male **(A)** and female **(B)**. Models are adjusted for age, gender, race, education level, marital status, family poverty-to-income ratio, smoking, alcohol, body mass index, total cholesterol, and fasting plasma glucose. Knots are the 25, 50, and 75th percentiles for metabolic equivalents.

The RCS model showed a non-linear negative dose–response correlation between PA (i.e., MET) and the OR for PAD (*p* for non-linearity = 0.0001) (Figure 1). The OR for PAD decreased as the PA increased, reaching the minimum at ~5,800 MET-min month^−1^ (OR = 0.5012, 95% CI = 0.5008–0.5015). After exceeding 5,800, the OR for PAD increased with excessive PA. Furthermore, the gender-stratified dose–response analysis (Figure 2) showed that PA and the incidence of PAD presented non-linear trends for both males and females similar to the total population (all *p* for non-linearity < 0.0001). For males, the curve decreases more sharply than that of females, and there is no obvious plateau. However, for females, the curve decreases more slowly reaching the minimum at ~5,800 MET-min month^−1^. After exceeding 5,800 MET-min month^−1^, the OR starts to increase. These findings suggest that males can do more PA, which can benefit their ABI, but for females, proper amount of PA is important, and excessive exercise may reduce the protective effects of PA.

### Subgroup Analyses of Physical Activity Patterns

We further analyzed the PA patterns of each group, we found that a higher proportion (Table 2) of individuals had no vigorous-intensity PA in the PAD group compared with individuals without PAD (*p* < 0.001). Next, we divided all the participants into two groups by the ratio of MET of vigorous/moderate (V/M) intensity PA. The V/M ratio for participant without vigorous PA in the past 1 month was 0 and >0 for participant with vigorous PA. The ORs and 95% CIs are listed in Table 4. The univariate analysis showed that compared with the V/M = 0 group, the OR of PAD for the V/M > 0 group was 0.422 (95% CI = 0.420–0.424). After adjusting for confounding factors, the OR was 0.667 (95% CI = 0.663–0.670) (all *p* < 0.001). These results indicated that proper vigorous PA could benefit peripheral artery health.

**Table 4:** Odds ratio (OR) of different patterns of physical activity for PAD.

**Variable**	**Univariate**		**Model I** ^**a**^		**Model II** ^**b**^	
	**OR (95%CI)**	***P*-value**	**OR (95%CI)**	***P*-value**	**OR (95%CI)**	***P*-value**
Vigorous/Moderate					
=0	1.000		1.000		1.000	
>0	0.422 (0.420–0.424)	<0.001	0.609 (0.605–0.612)	<0.001	0.667 (0.663–0.670)	<0.001
*P* for trend		<0.001		<0.001		<0.001

a *Adjust for age, sex, race, education level, and marital status*.

b *Adjust for age, sex, race, education level, marital status, family PIR, smoking, drinking alcohol, BMI, total cholesterol, and fasting plasma glucose*.

*OR, odds ratio; OR <1.0 means lower likelihood for PAD; CI, confidence interval; PIR, poverty-to-income ratio; BMI, body mass index*.

*Considered statistically significant p < 0.05.

## Discussion

This study found that PAD was presented in 6.2% participants, 5.6% of males and 6.9% of females in NHANES 1999–2004. Individuals with PAD showed a lower level of PA than those without PAD. In logistic regression analysis, PA was an independent protective factor for PAD. Furthermore, to our knowledge, there is no former study using RCS, to investigate the dose–response relationship between PA and incidence of PAD. The dose–response analyses after adjusting for confounders showed that PA levels were inversely associated with the incidence of PAD in all of the participants. For females, the OR of PAD decreased as PA increased before OR reaching minimum, as excessive PA reduced the protective effect. However, there was no extremum found in this study for males. This gender difference for PAD has been reported in previous studies. Wilson et al. found that women with low lifetime recreational activity were at greatest risk of PAD (22). It might explain the significant steeper decline in the first half of the RCS curve in the female group than that of the male group (Figure 2). This phenomenon suggests that moderate exercise has a more significant protective effect on the development of PAD in females. In addition, female gender was an independent risk factor for the development of PAD (21). Kamdem et al. also found by a cross-sectional study that baseline ABI was higher in males than females for both legs in healthy adults (23). These studies suggest that there may be inherent characteristics in females that contribute to greater susceptibility to PAD diagnosis. This could explain the upper limit of the protective effect of PA on PAD in females in our research when PA exceeded 5,800 MET-min month^−1^ (Figure 2). Further studies need to be done to explain the gender difference.

Next, by analyzing the patterns of PA in each group, we found a higher proportion of individuals who did vigorous-intensity activities in non-PAD group than that of the PAD group. In logistic regression analysis, higher proportion of vigorous-intensity activities is also a protective factor for PAD. The intrinsic mechanism cannot be determined from this study, but there are several physiological possibilities. Vigorous-intensity activities may enhance endothelial function, upregulating vasoprotective molecules such as superoxide dismutase, nitric oxide synthase, and downregulating the expression of vascular inflammation molecules (24, 25). In addition, PA in mice increases the release of endothelial progenitor cells, inhibits neointima formation after balloon injury, and promotes angiogenesis (26). In this study, the other risk factors for PAD were elder age, smoking, and fasting plasma glucose. This is consistent with previous studies suggesting that cigarette smoking and diabetes are significant risk factors for PAD (27, 28).

Other studies have explored the relationship between PA and the incidence of PAD. In the Cardiovascular Health Study, the incidence of PAD, as diagnosed by ABI <0.9, increased from low to medium to high exercise intensity categories in both genders (29). Another prospective research examined the relationship between exercise training and ABI in patients without PAD but at high risk due to the presence of type 2 diabetes (22). Over a 6-month intervention, ABI increased modestly in exercise group while it decreased in control group (*p* for interaction = 0.001). In more general population, there are limited studies that PA may reduce incidence of PAD (29, 30). Though these studies cannot evaluate temporality, they are consistent with our findings.

However, the results of some previous studies do not support our conclusions. A Cochrane Review of exercise therapy for intermittent claudication showed that physical training did not affect ABI in seven studies with a pooled effect of −0.01 (95% CI = −0.05–0.04) (31). Besides, several long-term follow-up studies have found no association with PA and ABI (32, 33), but these studies are based on patients with symptomatic PAD or intermittent claudication. It is possible that exercise training can be more effective for improving ABI earlier in the atherosclerosis process before the diagnosis of PAD or a more profound reduction of blood flow in legs.

The strengths of our study include the inclusion of a representative population from a nationwide survey on non-institutionalized people. Logistic regression and RCS models were used to evaluate the relationship between PA and incidence of PAD, and stratification was performed for gender. By using the RCS model, dose–response relationship was ideally evaluated without jumping directly from one interval to another, avoiding the subjectivity of traditional regression methods. In this study, we innovatively identified a mirrored “J-shaped” relationship between PA and PAD risk by the RCS method, especially for females (Figure 2), revealing that neither too low nor high PA may be optimal for PAD prevention. The determination of these non-linear dose–response relationship helps public health leaders to develop medical policies. The finding of this paper also inspires further basic research on the causal mechanisms underlying the “J-shaped” trajectory.

Our study has some limitations. First, its cross-sectional design meant that this study was not possible to determine causality, and hence, it did not rule out the possibility that patients with PAD changed their PA after the condition. We excluded persons with low cardiovascular fitness level who are likely to have cardiovascular diseases limiting physical activity. Second, the number of participants in NHANES 1999–2004 is 31,126, but most individuals have missing data of the interested factors. Thus, this study has a limited population of 1,370 subjects. Third, the PA was based on self-reporting questionnaires, which might be affected by memory bias, but overall, the database analysis included a representative population and provided good data to facilitate clinical research.

## Conclusion

There is a significant inverse dose–response relationship between PA and the incidence of PAD. When PA increased to 5,800 MET-min month^−1^, the prevalence of PAD in the overall population decreased by around 50%, while increases in PA may benefit males but reduce the protective effect of PA in females.

## Data Availability Statement

The original contributions presented in the study are included in the article/supplementary material, further inquiries can be directed to the corresponding author/s.

## Ethics Statement

The studies involving human participants were reviewed and approved by National Center for Health Statistics Ethics Review Board. The patients/participants provided their written informed consent to participate in this study.

## Author Contributions

C-JQ: data curation (lead), methodology (lead), visualization (lead), and writing original draft (lead). X-NL: data curation (supporting) and writing original draft (supporting). Y-BZ: methodology (supporting) and writing editing (supporting). JF: methodology (supporting) and writing review (supporting). L-QT: visualization (supporting) and methodology (supporting). C-YS: conceptualization, funding acquisition, and writing-reviewing and editing (lead). All authors contributed to the article and approved the submitted version.

## Funding

This work was financially supported by a grant from the National Natural Science Foundation of China (81870350).

## Conflict of Interest

The authors declare that the research was conducted in the absence of any commercial or financial relationships that could be construed as a potential conflict of interest.

## Publisher's Note

All claims expressed in this article are solely those of the authors and do not necessarily represent those of their affiliated organizations, or those of the publisher, the editors and the reviewers. Any product that may be evaluated in this article, or claim that may be made by its manufacturer, is not guaranteed or endorsed by the publisher.
